# Immunopotentiation by Lymph-Node Targeting of a Malaria Transmission-Blocking Nanovaccine

**DOI:** 10.3389/fimmu.2021.729086

**Published:** 2021-08-27

**Authors:** Gregory P. Howard, Nicole G. Bender, Prachi Khare, Borja López-Gutiérrez, Vincent Nyasembe, William J. Weiss, Jerry W. Simecka, Timothy Hamerly, Hai-Quan Mao, Rhoel R. Dinglasan

**Affiliations:** ^1^Department of Biomedical Engineering, Johns Hopkins University School of Medicine, Baltimore, MD, United States; ^2^Institute for NanoBioTechnology, Johns Hopkins University, Baltimore, MD, United States; ^3^Emerging Pathogens Institute, Department of Infectious Diseases & Immunology, College of Veterinary Medicine, University of Florida, Gainesville, FL, United States; ^4^Department of Pharmaceutical Sciences and UNTHSC Preclinical Services, University of North Texas System College of Pharmacy, University of North Texas Health Science Center, Fort Worth, TX, United States; ^5^Department of Materials Science and Engineering, Whiting School of Engineering, Johns Hopkins University, Baltimore, MD, United States; ^6^Translational Tissue Engineering Center, Johns Hopkins University School of Medicine, Baltimore MD, United States

**Keywords:** nanoparticle, malaria transmission-blocking vaccine, humoral immune response, lymph node, vaccine, biodegradable, trafficking

## Abstract

A successful malaria transmission blocking vaccine (TBV) requires the induction of a high antibody titer that leads to abrogation of parasite traversal of the mosquito midgut following ingestion of an infectious bloodmeal, thereby blocking the cascade of secondary human infections. Previously, we developed an optimized construct UF6b that elicits an antigen-specific antibody response to a neutralizing epitope of Anopheline alanyl aminopeptidase N (AnAPN1), an evolutionarily conserved pan-malaria mosquito midgut-based TBV target, as well as established a size-controlled lymph node targeting biodegradable nanoparticle delivery system that leads to efficient and durable antigen-specific antibody responses using the model antigen ovalbumin. Herein, we demonstrate that co-delivery of UF6b with the adjuvant CpG oligodeoxynucleotide immunostimulatory sequence (ODN ISS) 1018 using this biodegradable nanoparticle vaccine delivery system generates an AnAPN1-specific immune response that blocks parasite transmission in a standard membrane feeding assay. Importantly, this platform allows for antigen dose-sparing, wherein lower antigen payloads elicit higher-quality antibodies, therefore less antigen-specific IgG is needed for potent transmission-reducing activity. By targeting lymph nodes directly, the resulting immunopotentiation of AnAPN1 suggests that the de facto assumption that high antibody titers are needed for a TBV to be successful needs to be re-examined. This nanovaccine formulation is stable at -20°C storage for at least 3 months, an important consideration for vaccine transport and distribution in regions with poor healthcare infrastructure. Together, these data support further development of this nanovaccine platform for malaria TBVs.

## Introduction

Malaria continues to be a persistent threat to the world population with more than 229 million individuals infected worldwide and 409,000 deaths recorded in 2019 (1). Transmission-blocking vaccines (TBVs) have long been considered as an ideal way to control, if not eliminate, the disease (2–4), and recent studies suggest that TBVs would be acceptable in malaria-endemic settings (5). Transmission of *Plasmodium* parasites though the female *Anopheles* mosquito vector is contingent on the development to the ookinete stage, which must traverse through the mosquito midgut to form an oocyst and develop into sporozoites that then access the salivary glands, leading to subsequent human infections during blood feeding. TBVs disrupt this obligatory step of midgut traversal in the parasite life cycle, reducing the number of infectious vectors and the cascade of secondary infections in the human population. The mosquito midgut protein, Anopheline alanyl aminopeptidase N (AnAPN1), is the leading mosquito-based TBV candidate, with previous studies demonstrating up to 100% transmission-blocking (T-B) activity against *Plasmodium falciparum* by rabbit polyclonal and mouse monoclonal antibodies (6, 7). Parallel structure-based studies identified the T-B epitopes as peptides 7 and 9 (6). To focus the immune response to the key T-B epitopes we developed a new, purification tag-free AnAPN1 construct, UF6b, containing two copies of peptides 2-9 connected by a glycine linker (8). This new construct can be produced at scale and was shown to be highly immunogenic in outbred CD1 mice immunized intramuscularly (*i.m.*) with UF6b formulated with the human-safe adjuvant Glucopyranosyl Lipid Adjuvant in a liposomal formulation with saponin QS21 (GLA-LSQ), eliciting a focused peptide 9 specific response resulting in potent T-B antibodies (6, 8).

Nanoparticle-based vaccines in recent years have been designed to further improve vaccine efficacy, target key lymphoid tissues, and increase persistence of vaccine antigen and adjuvant cues to achieve desired humoral and cell-mediated immune responses (9). Nanoparticles (NPs) are safe for human use and can improve vaccine stability and effectiveness by protecting antigens from proteolytic digestion, enabling and manipulating antigen processing by antigen presenting cells, and by exhibiting controlled antigen availability *via* release kinetics (10). To generate an efficient immune response, it is essential for the antigen to localize to the lymph nodes where naïve antigen presenting cells (APCs) are present at high density (11–13). Targeting naïve APCs in these tissues enables APC activation and presentation of antigenic cues to T- and B-cells, leading to germinal center formation and production of high affinity antigen-specific antibodies (13–15). To access these key immune cell populations in the lymph node, administration of antigen and adjuvant cues by subcutaneous (*s.c.*) or intradermal (*i.d.*) injection utilizes the lymphatic drainage system that constantly drains fluid and macromolecules from the interstitial cellular spaces at a flow rate of 0.1–1 μm/s to the local draining lymph nodes (16–18). NP size is a determining factor in their localization to the lymph nodes. It has previously been demonstrated that synthesizing NPs in the size range of viruses enables their quick localization to the lymph nodes after *s.c.* or *i.d.* administration, making these vaccines more efficient at eliciting an immune response (19–21). In contrast, NPs larger than 100 nm will generally remain at the injection site (22–25). Therefore, an ideal TBV NP vaccine would have uniform, small (<50 nm) sizes that allow for targeting of key APC populations and induction of a durable humoral immune response.

Biodegradable polyesters including poly(lactic acid) (PLA), poly(glycolic acid) (PGA), poly (ϵ-caprolactone) (PCL) and their co-polymers have a long precedence and proven safety track record in the clinic making these materials the preferred choice for NPs in clinical applications (22, 26). Synthetic oligonucleotides (ODNs) like CpG have been shown to boost humoral and cellular vaccine specific immune responses *via* activation of cells that express Toll-like receptor 9 (27). Class B CpG-1826 has been shown to activate cytotoxic T cells *via* lymph node localizing NP vaccine (28). CpG-1018 immunostimulatory sequence (ISS), a Class B CpG with a full phosphorothioate backbone for enhanced stability against enzymatic degradation, was approved by the FDA in 2016 for a Hepatitis B vaccine (HEPLISAV-B™) (29). As the first approved use of CpG ODNs in the clinic, this vaccine demonstrated an excellent safety profile with few adverse effects and increased vaccine efficacy in populations (*e.g.*, diabetics, elderly) that typically do not achieve protection using previous Hepatitis B vaccines. The CpG-1018 construct exhibits strong antigen dose sparing at high adjuvant:antigen ratios (*e.g.*, 150:1 CpG-1018:antigen ratio) and allowed for a reduction in dosing schedule from a typical three shot schedule (0, 6, 12 months) as seen in previous Hepatitis B vaccines (*e.g.*, Engerix-B, Twinrix) to a two shot schedule (0, 1 month) while achieving higher seroprotection as defined as ≥10 mIU/mL anti-HB serum antibodies, a strong correlate of protection (30). Given the proven record of CpG-1018 in the clinic for induction of high antigen-specific antibody titer, we selected CpG-1018 ISS as our adjuvant for further study with our UF6b AnAPN1 immunogen construct. Furthermore, previously published studies with and without model antigen ovalbumin have provided evidence to further test the efficiency of the immune response to the UF6b antigen with 30 nm NPs synthesized using flash nanoprecipitation (FNP) which produced well controlled size and narrow-size distribution particles (20, 21).

Here, we describe the formulation, lyophilization optimization and stability, lymph node targeting, and functional immune response of outbred CD1 mice to a matrix of Nano-UF6b and Nano-CpG (1018-ISS) vaccine co-formulations in comparison to the benchmark formulation of UF6b with AddaVax™, a squalene-based oil-in-water nano emulsion adjuvant, and summarize our insights into how formulations of this novel, disease-agnostic nanovaccine platform can immuno-potentiate existing as well as next generation malaria TBV targets in the future.

## Materials and Methods

### Materials

All organic solvents and hydrophobic IR-780 iodide (dye content ≥95%) were purchased from Sigma-Aldrich (St. Louis, MO, USA). PLGA_20K_-*b*-PEG_3K_-Maleimide (PLGA-*b*-PEG-Mal) and PLGA_20K_-*b*-mPEG_3K_ (PLGA-*b*-mPEG) polymers were both purchased from PolySciTech^®^ (West Lafayette, IN, USA). Spectra/Por™ 3 RC 3.5 kDa MWCO dialysis tubing was purchased from Repligen™ (Waltham, MA, USA). 100 kDa MWCO Amicon centrifugal filters were purchased from Millipore Sigma (Burlington, MA, USA). CpG-1018 ODN ISS (Sequence: 5’-thiol S-S C6-TGACTGTGAACGTTCGAGATGA-3’) with a phosphorothioate (PS) backbone was synthesized by TriLink Biotechnologies (San Diego, CA USA). UF6b peptide construct (Sequence: MCDLHLRTEIHRNERTFTGTVGIQLQVVQATDKLVMHNRGLVMSSAKVSSLPNGVTGAPTLIGDVQYSTDTTFEHITFTSPTILQPGTYLLEVAFQGRLATNDDGFYVSSYVADNGERRYLAGSGGGGSGGGGSGDLHLRTEIHRNERTFTGTVGIQLQVVQATDKLVMHNRGLVMSSAKVSSLPNGVTGAPTLIGDVQYSTDTTFEHITFTSPTILQPGTYLLEVAFQGRLATNDDGFYVSSYVADNGERRYLACGGSG) was synthesized by CellFree Sciences Co (Tsurumi-ku, Yokohama JPN).

### PLGA-*b*-PEG Nanoparticle Fabrication

NPs were generated using a three-inlet, confined impinging jet FNP device. Two inlets contained distilled, deionized (DDI) water. The third inlet contained 1 mL of 10 mg/mL PLGA_20K_-*b*-mPEG_3K_ with 78.5 mol % PLGA_20K_-*b*-mPEG_3K_ and 21.5 mol % PLGA_20K_-*b*-PEG_3K_-Mal dissolved in tetrahydrofuran (THF). NPs of defined size were generated by modulating volumetric flow rates of the three input inlets using a NE-4000 Programmable 2 Channel Syringe Pump (New Era Systems, Inc., Farmingdale, NY, USA). For 30 nm NPs, a flow rate of 22 mL/min was used for all three inlets. The NPs generated through the device were collected in a water bath such that the total organic solvent was less than 10% v/v. The collected NPs were dialyzed against 4 L of DDI water in a 3.5-kDa MWCO dialysis membrane at 4°C with dialysate changes every 6 h for 18–24 h.

#### Preparation of UF6b Peptide and CpG-1018 ISS for Conjugation to NPs

To remove the 5’ C6 thiohexyl modification from the CpG-1018 ISS and to present a reactive thiol terminus for NP conjugation, 400 µL of 0.1 M TCEP in sterile RNase free water was added directly to the lyophilized thiolated CpG-1018 for 1 h to reduce the thiol groups under intermittent vortexing. After 1 h, 50 µL of 3 M Sodium Acetate was added and vortexed. 1.5 mL of absolute ethanol was added, the solution vortexed, and stored at -20°C for 20 minutes. The sample was then centrifuged at 12,000 rpm for 10 min. The ethanol was decanted and allowed to air dry in a sterile cell culture cabinet. The CpG-1018 ISS pellet was dissolved in 200 µL of sterile RNase free water and the sample concentration was obtained by measuring the absorbance at 260 nm. To reduce the terminal thiol(s) on the UF6b peptide, the UF6b peptide was treated with 10× molar excess of TCEP in DI water for 1 h at room temperature (RT) under intermittent vortexing. The excess TCEP was removed by dialysis of the UF6b peptide using a 3.5 kDa MWCO dialysis membrane and degassed 4L 0.1×PBS with 2 mM EDTA at pH 6.2–6.5 with changes every 3 h.

#### Conjugation of UF6b and CpG to NPs and Lyophilization

NPs during the final DDI water dialysis step were transferred to 4 L of 0.1×PBS buffer with 2 mM EDTA at pH 6.2-6.5, with another dialysis change in the same buffer after 6 h. The NP solution at a concentration of 300 µg/mL was collected and then reacted with either UF6b-SH or CpG-SH at a maleimide:SH ratio of 1:1 for 16 h at 4°C. The reaction pH was tuned to 6.2-6.5 after components were mixed by adding NaOH. NPs were then collected, washed with DDI water to remove unreacted UF6b or CpG, and concentrated at 400×g at 5 min intervals using a 100 kDa MWCO centrifugal filter and a Sorvall RT1 Centrifuge (Thermofisher Scientific, Waltham, MA, USA) until the desired concentration of UF6b-NP or CpG-NP was reached as measured by Pierce™ microBCA Protein Assay (Thermofisher Scientific) and UV-Vis using a NanoDrop 1000 Spectrophotometer (Thermofisher Scientific), respectively. UF6b-NP and CpG-NP groups were mixed directly to achieve various desired UF6b:CpG ratios and doses.

#### Lyophilization and Stability Studies of Conjugation and Unconjugated Nanoparticles

To determine the optimal lyophilization and storage conditions of NPs, unconjugated NPs were screened with a variety of concentrations of cryoprotectants. First, NPs at the final desired concentration were resuspended in either 1% w/v xylitol with 0.5% w/v mannitol, 2% w/v xylitol with 1% w/v mannitol, 10% w/v trehalose, 20% w/v trehalose, 10% w/v sucrose, or 20% w/v sucrose. NPs were either snap-frozen in liquid nitrogen or slowly frozen using a Mr. Frosty™ Freezing Container (Thermofisher Scientific) at -80°C for 6 h, and then thawed at RT before measuring the NP size. Sugars and concentrations that conferred protection with freeze/thaw cycles were selected for final study. Second, NPs were again frozen either slowly or snap-frozen as detailed above and then lyophilized using a FreeZone Triad Benchtop Freeze Dryer (LABCONCO, Kansas City, MO, USA) for 48–72 h. NPs were reconstituted and vortexed for 10 sec. Reconstituted NP size was then immediately measured by dynamic light scattering using a Malvern Zetasizer Nano ZS (Malvern, Worcestershire, UK). The most stable formulation at the lowest cryoprotectant concentration was then chosen for the vaccination studies. Stability is here defined as minimal size change from pre- to post-lyophilization that preserves the NP size below 50 nm (number average) as measured by DLS.

#### Lyophilization of UF6b- and CpG-Nanoparticles Used for Vaccination Study

UF6b-NPs and CpG-NPs were mixed with 20% w/v sucrose cryoprotectant to yield the desired dose of antigen/adjuvant with a final concentration of 10% w/v sucrose. The NPs were aliquoted by 300 µL into sterile 2.0 mL screw-top tubes, frozen overnight using a Mr. Frosty in an -80°C freezer and lyophilized using a FreeZone Triad Benchtop Freeze Dryer. The lyophilized powder was stored in the -80°C freezer until use. To reconstitute the NPs, 300 µL of DI water was added and vortexed for 10 sec. NPs were then immediately administered to mice.

### Physical Characterization of Nanoparticles

The NP before and after UF6b- or CpG-conjugation were diluted to 100 µg/mL in reference to polymer weight in 10 mM HEPES (pH 7.2, containing 10 mM NaCl) and the hydrodynamic diameter and zeta potential were both measured using a Malvern Zetasizer Nano ZS. The morphology and dry size of each NP was determined by transmission electron microscopy (TEM) using a Tecnai FEI-12 electron microscope (FEI Company, Hillsboro, OR, USA). Samples were diluted and 20 μL aliquots absorbed onto Electron Microscopy Sciences ionized nickel grids covered with carbon films for 30 min. The NP suspension was removed by blotting using Whatman filter paper or Kimwipe™. The samples were then stained with 2% w/v uranyl acetate for 45 sec. The grids were blotted to remove excess negative stain and allowed to dry in a chemical hood prior to imaging.

### *In Vivo* Trafficking and Whole Body Imaging

NPs were fabricated as above with the addition of 24.5 mol% IR-780 iodide dye dissolved in THF and mixed with the polymer (Sigma Aldrich) to allow for near infrared (NIR) whole body imaging. The treatment groups were generated using the above conjugation protocol to first generate UF6b-NP and CpG-NP which were then mixed together at the appropriate ratios to make 25UF-0C, 25UF-0.25C, 0UF-5C, and unconjugated maleimide NP (Mal-NP) control. Female CD1-IGS outbred mice (6-8 weeks) from Charles River Laboratories (Germantown, MD, USA) were then injected (*s.c.*) with 50 µL of nanoparticle suspension in 10% w/v sucrose 1 cm below the tail-base. Mice were sacrificed at 3 or 24 h to isolate the major reticuloendothelial system (RES) organs and imaged. Mice without the RES were then imaged to demonstrate drainage to the major draining lymph nodes. The IR780 Iodide dye signal encapsulated within the PLGA-b-PEG NPs were then quantified using Pearl Image Studio Lite Ver 5.2 to determine the percent injected dose.

### Vaccination

Female CD-1-IGS outbred mice (6-8 weeks) from Charles River Laboratories (Germantown, MD, USA) were divided into nine immunization groups outlined in **Table 1**, with six mice per group. Mice were immunized *s.c.* with 50 µL of NP suspension in 10% (w/v) sucrose buffer at 1 cm from lateral tail base in a prime and boost (boost on day 28) regimen.

**Table 1:** Antigen/adjuvant nano-constructs used for mouse immunizations (*s.c.*).

Group Name	Test Material	UF6b:CpG Ratio	Dose UF6b (µg)	Dose CpG (µg)
25UF-0C	UF6b-NP	N/A	25	0
5UF-0C	UF6b-NP	N/A	5	0
0UF-5C	CpG-NP	N/A	0	5
5UF-0.05C	UF6b-NP/CpG-NP	100:1	5	0.05
5UF-0.5C	UF6b-NP/CpG-NP	10:1	5	0.5
5UF-1C	UF6b-NP/CpG-NP	5:1	5	1
25UF-0.25C	UF6b-NP/CpG-NP	100:1	25	0.25
Blank	Empty NP (Negative control)	N/A	0	0
5UF-AddaVax™	UF6b-NP + AddaVax™ (Positive control)	N/A	5	0

Starting on day 0 (day of priming dose) and every two weeks thereafter, sera was collected for enzyme-linked immunosorbent assay (ELISAs) and standard membrane feeding assays (SMFAs). Mice were sacrificed at 10 weeks post-prime and blood collected *via* cardiac puncture.

A replicate study was completed as described above using a separate cohort (SC2) of mice.

### Enzyme Linked Immunosorbent Assays

The ELISAs were performed as previously described (8). Briefly, Nunc Maxisorp 96-well ELISA plates (Fisher Scientific, Waltham, MA, USA) were incubated overnight at 4°C with 1 μg/mL UF6b antigen in 0.1×PBS (pH 7.2). After three washes with PBS-Tween 20 (0.05%) (PBST20), the plates were blocked for 1 h at RT with 1% bovine serum albumin (BSA) in 1×PBS. Serum samples were first diluted to 1:10^2^ and 1:10^3^ then further serially two-fold diluted to 1:64000 in 0.5% w/v BSA in 1×PBS. After discarding the BSA and drying the plates, 100 μL of each of the eight sample dilutions were added to each well in triplicate and incubated 1 h at RT. Plates were washed three times with PBST20. Then, 100 μL of a horseradish peroxidase (HRP)-conjugated goat anti-mouse IgG (H+L) (Bio-Rad, Hercules, CA, USA) diluted 1:5000 in 0.5% w/v BSA was added to each well and incubated for 1 h at RT. Plates were washed three times with PBST20 and developed by adding 100 µL of KPL TMB Microwell Peroxidase Substrate (Bio-Rad) to each well. Development was stopped after 5 min by the addition of 100 μL of 1M H_3_PO_4_. A BioTek Synergy™ HTX Multi-Mode Microplate Reader (Winooski, VT, USA) was used to read the OD values at 570 nm and 450 nm. The OD values at 570 nm were subtracted from the 450 nm values to account for background.

The isotype of the UF6b-specific elicited antibody was determined using a Mouse Typer Isotyping Panel (Bio-Rad) as previously described (8). Briefly, plates were coated and blocked as described above. The mouse sera was diluted (1:1000 in 0.5% w/v BSA) and incubated for 1 h at RT. After washing five times with PBST, the isotyping panel was added, washed again with PBST for five times, and then HRP-conjugated goat anti-rabbit IgG (H+L) (Bio-Rad) was added. Plates were read as described above.

### Mosquito Colony

*Anopheles gambiae* (Keele) was used in all experiments. Mosquitoes were reared under standard insectary conditions of 27°C, 80% relative humidity, and 12 h:12 h light:dark cycle. Eggs were hatched in Milli-Q water supplemented with 0.02% yeast slurry. The emerging larvae were reared in plastic trays (25 cm long × 20 cm wide × 14 cm high) at a density of 300–400 larvae per tray and provided a daily ration of 1 g koi fish food per tray/day. Pupae were collected and put in holding cages for emergence. Emerged adults were fed *ad libitum* on 10% sucrose solution. For all experiments, 5-to-6-day old adult females were used.

### Standard Membrane Feeding Assays

*P. falciparum* NF54 parasites were cultured following standard conditions at 37 °C in hypoxic conditions. Briefly, parasites were maintained with O+ human erythrocytes at 4% hematocrit in RPMI 1640 media supplemented with hypoxanthine and 10% O+ heat-inactivated human serum (HIHS). Gametocytes cultures were seeded at 0.5% asexual parasitemia and were continuously cultured with daily media changes. Gametocytes were harvested 17 days after initiation and packed infected red blood cells were diluted to 30% hematocrit and 1% stage V gametocytemia with HIHS and uninfected erythrocytes. Infective blood was mixed with control (PBS) or total IgG purified from pooled sera of mice immunized with UF6b prior to delivery directly into water-jacketed membrane feeders maintained at 37°C *via* a circulating water bath. Total IgG was purified by Protein A/G Magnetic Agarose Beads (Thermo Fisher Scientific) from pooled of immune sera. The positive control used was pooled IgG from UF6b-AddaVax immunized mice, as this has been shown to elicit a polyclonal antibody response and would be an appropriate comparator for IgG isolated from the nanovaccine-treated mouse cohorts. Since pre-immune IgG from the immunized mice is limiting and considering that naïve, purified mouse IgG has never been shown to have intrinsic transmission-blocking activity in the SMFA, we used as a negative control pooled, naïve human sera, as described previously (31–33). The final concentration of total IgG in 300 μL total volume of infective blood was 750 μg/mL for each technical replicate. The UF6b- specific IgG antibody concentration was determined by first purifying pooled pre-immune and immune serum with UF6b conjugated Dynabeads™ M-270 and then measuring using the Easy-Titer™ Mouse IgG assay kit following the manufacturer’s instructions (Thermo Fisher Scientific). Female *An. gambiae* (Keele) (n = 50) mosquitoes were starved overnight and allowed to feed for 30 min. Unfed mosquitoes were collected and discarded after 12–18 h post-feed. Midguts were dissected, and oocysts were enumerated by microscopy 8 days post-blood feeding. Three independent experiments were performed for each immunization group.

### Statistical Analysis

Data are shown as either mean ± standard deviation or mean ± standard error mean as noted in the figure captions. All statistical analyses were performed with GraphPad Prism v. 9.2 software package. SMFA data were analyzed by non-parametric Kruskal-Wallis test with Dunn’s multiple comparison *post-hoc* test to determine differences between groups. All other data was analyzed using an unpaired *t* test for direct comparisons or an ordinary one-way ANOVA followed by a Tukey’s or Dunnett’s post-test, unless otherwise noted. The values were considered significantly different at *p* < 0.05. Statistics on graphs were displayed as not significant (ns) *p >* 0.05, *p < 0.05, **p < 0.01, ***p < 0.001, ****p < 0.0001 with *α* = 0.05.

## Results

**Figure 1:**
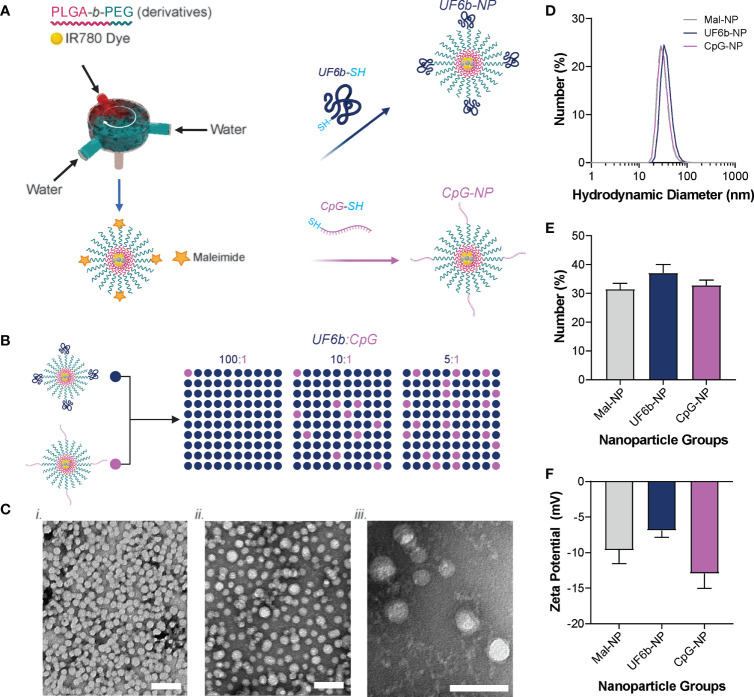
Nanoparticle conjugation scheme. **(A)** PEG-*b*-PLGA NPs were produced by flash nanoprecipitation (FNP) and then conjugated with thiolated UF6b or CpG oligodeoxynucleotide containing immunostimulatory sequence (ODN ISS) 1018. **(B)** The UF6b-NP and CpG-NP were then mixed together in different mass ratios to yield the NPs used for the vaccination study. **(C)** Transmission Electron Microscopy of the Mal-NP, UF6b-NP, and CpG-NPs. Scale bars = 50 nm. **(D)** Size distribution of each NP population, **(E)** the mean number-weighted hydrodynamic diameter of each NP population, and **(F)** zeta potential of each NP population measured in 10 mM NaCl and 10 mM HEPES (pH 7.4). Error bars indicate standard deviation of four replicates.

NPs generated using the FNP method and subsequent conjugation with CpG-1018 and UF6b construct are reproducible from batch to batch (**Figure 1** and **Supplemental Table 1**). The densities of UF6b antigen and CpG-1018 adjuvant were selected based off our previously published studies (20, 21). The literature demonstrates that lower antigen density is more immunogenic than higher density; in addition, lower CpG adjuvant density skews towards a Th2-type antibody response (**Supplemental Table 2**) (34, 35). NPs off the FNP device and after dialysis had a number average size of 31.45 ± 1.96 nm with a zeta potential of -9.66 ± 1.89 mV. After conjugation with CpG-1018-SH overnight, the NP size grew slightly to a number average size of 32.80 ± 1.81 nm and became more negative with a zeta potential of -12.95 ± 2.05 mV, suggesting successful conjugation of the CpG construct onto PLGA-*b*-PEG NPs (CpG-NPs). Similarly, after conjugation with UF6b-SH overnight, the NP size grew to a number average size of 37.13 ± 2.93 nm and became more neutral in charge with a zeta potential of -6.88 ± 0.95 mV, likewise suggesting successful conjugation of the UF6b construct onto PLGA-*b*-PEG NPs (UF6b-NPs). Similar results in size, PDI, zeta potential, and conjugation efficiency were observed over four independent batches of UF6b and CpG NP preparations (**Supplemental Table 1**).

A NP vaccine formulation that is shelf-stable is of paramount importance with a preference for formulations that can withstand minimal cold transport conditions. To fabricate such a NP vaccine formulation, we screened a variety of cryoprotectants including sucrose, trehalose, and xylose mixed with mannitol for lyophilization. We found that only the sucrose cryoprotectant maintained the NP size before and after a flash freeze/thaw test (**Supplemental Figure 1A**). All other cryoprotectants failed to protect in the flash freeze/thaw test and so were not selected for the final lyophilization test. Both the 10% and 20% w/v sucrose groups then showed protection of NP size post-lyophilization with minimal NP size and PDI change. Furthermore, the 10% and 20% w/v sucrose maintained the NP size, using best-performing formulation 25UF-0.25C as a test case, after 1 and 3 months in -20°C storage (**Supplemental Figure 1B**). These results demonstrate that these NP formulations are stable over a 3-month period in -20°C storage.

We have previously demonstrated that the PLGA-*b*-PEG NP delivery system efficiently drains to the local draining lymph nodes in a size-dependent manner without the conjugation of antigenic or adjuvant cues (20). To determine that similar drainage would occur using UF6b-NP and CpG-NP mixed together at desired antigen:adjuvant ratios, we administered four different candidate groups (25UF-0C, 25UF-0.25C, 0UF-5C, and control unconjugated maleimide-NP) that were fluorescently labeled with NIR dye IR780 Iodide to CD1-IGS mice and acquired whole body images at 3 and 24 h. As expected, all NP groups efficiently drained to the lymph nodes over 3 h and had retention in the draining lymph nodes at 24 h (**Supplemental Figure 2**). These results show that this NP vaccine platform with co-delivered antigenic and adjuvant cues targets the lymph node after *s.c.* administration.

**Figure 2:**
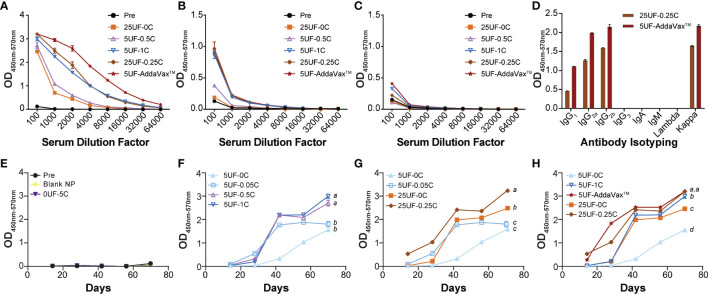
Specificity and antibody response kinetics to UF6b at day 70. **(A)** Antibody-specific titer to whole UF6b peptide construct. **(B)** Antibody-specific titer to peptide 9 sequence. **(C)** Antibody-titer specific to peptide 7 sequence. **(D)** Antibody isotyping for 25UF-0.25C and 5UF-AddaVax™. **(E–H)** Antibody kinetics over 70 days. The 1:100 dilution from the UF6b-specific antibody ELISA data was plotted over 70 days for each treatment. The data was grouped for comparison. **(E)** Control samples. **(F)** Doping in of CpG-NP for 5 µg UF6b-NP dose. **(G)** Comparison of 1:100 UF6b:CpG ratio for 5 µg and 25 µg UF6b doses. **(H)** Demonstrating how CpG doping allows for increase in antibody titer and kinetic, but neither 5 µg nor 25 µg UF6b-NP dose with CpG-NP is as potent as 5 µg UF6b with AddaVax™ adjuvant. Pooled sera from 6/mice per group were analyzed. Data points represent the mean of triplicate wells. Error bars indicate SEM of triplicates, although in some cases the variation is tightly controlled, and the error bar is obscured by the data point. Treatments without a common letter were found to be statistically significant (α = 0.05) by ANOVA followed by Tukey’s test.

Previously we have shown that the UF6b antigen is highly immunogenic in CD1 outbred mice (8). Here we aimed to find the optimally immunogenic dose and ratio of UF6b-NPs to CpG-NPs. Mice were vaccinated subcutaneously at 1cm lateral of the tail base using a prime and boost regimen with doses on day 0 and day 28, respectively. Tail-snip bleeds were collected every two weeks post prime until the end of the study at day 70 to monitor the development of the UF6b antigen specific-IgG response by indirect ELISA (**Supplemental Figure 3**). We observed that of the groups tested, the positive control group, 5UF-AddaVax™, elicited the strongest and most rapid antibody response, while in groups containing UF6b-NPs and CpG-NPs, we found that 5UF-1C and 25UF-0.25C elicited the highest titers (**Figure 2**). The 25UF-0.25C and 5UF-AddaVax™ had similar Th1/Th2 humoral immune response profiles as assessed by IgG subclass analysis (**Supplemental Figure 4**). It was also observed that groups 5UF-0C and 25UF-0C, UF6b-NPs in the absence of an adjuvant, were slightly immunogenic (**Figures 2F–H**). Except where noted, differences in antibody titers at day 70 were found to be statistically significant (*p < *0.05).

We determined the ability of this vaccine modality to generate a peptide 9 specific antibody response and found that only the three groups with the highest UF6b titers also elicited peptide 9 specific responses with very little variation between the responses. No groups tested elicited a strong peptide 7 specific response. A second cohort replicate study was also conducted to determine the reproducibility of the NPs generated and the immune response elicited by the mice (**Supplemental Figures 5, 6**). In the replicate study we found some variation in the relative antibody responses between the groups. 5UF-AddaVax™ still gave the strongest response; however, there was an increase in titer in groups 25UF-0C, 5UF-0.05C, 5UF-0.5C and a decrease in groups 5UF-1C and 25UF-0.25C. Only 5UF-0.5C and 5UF-AddaVax™ appeared to elicit a strong peptide 9 specific response in the replicate study. There was no change in peptide 7 recognition, as has been previously shown in mouse studies (6, 8), indicating that although peptide 7 is predicted to be a Class II binding epitope in humans (36), this is not the case for mice. Variation in immune response is expected in outbred mouse models such as CD1-IGS. Lymph node targeting efficiency varies greatly from mouse to mouse with some having left v. right bias in drainage or little to no drainage as previously reported (20). This can be reflective of differences in lymphatics for individual mice or variations in experimental technique. This combination of lymphatic variation and the innate genetic variability in outbred mice models may explain this discrepancy in reproducibility.

**Figure 3:**
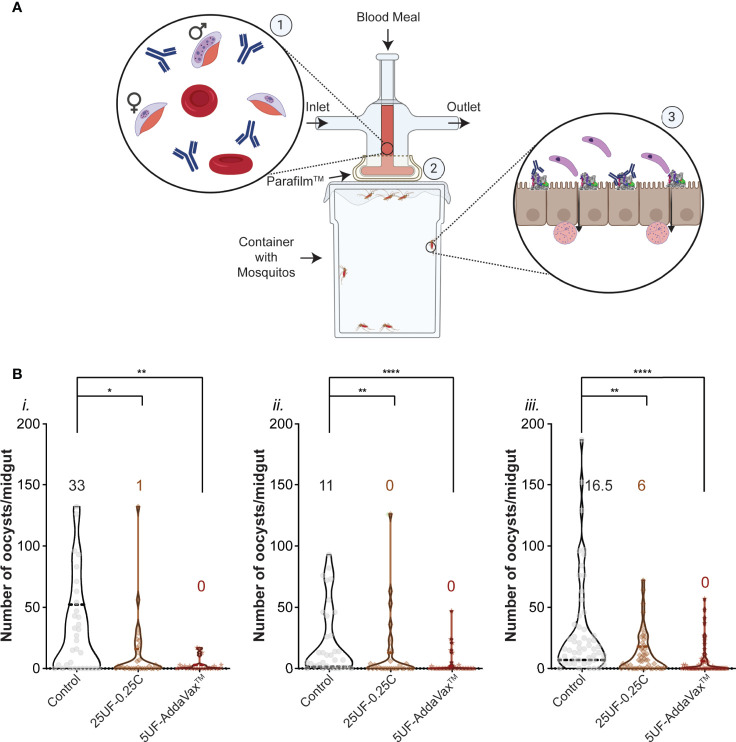
*Plasmodium falciparum* transmission-reducing activity of immune sera elicited by the UF6b nanovaccine. **(A)** Schematic of the Standard Membrane Feeding Assay (SMFA). ①Purified total IgG is added to a gametocytemic blood meal ② which is then fed through a membrane feeder to *Anopheles* mosquitoes. ③ Within the mosquito midgut, antibodies bind to AnAPN1 and block transmission of the ookinete (top) through the epithelium. Ookinetes that can bind to AnAPN1 pass through and form oocysts (bottom) which are then counted. Figure created with BioRender.com. **(B)** Three independent replicates (*i.*, *ii.*, *iii.*) of the SMFA assay using a feed of 750 µg/mL of purified IgG per group. Control is untreated gametocytemic blood. Overlay numbers are median values of oocysts/midgut for each group. Mosquito sample sizes for each control/test group from left to right: (*i.)* 32, 32, 23 (*ii.*) 33, 31, 34 and (*iii.*) 54, 53, 60. Dotted and bold lines correspond to the quartiles and median, respectively. Significance (α = 0.05) was calculated by Kruskal-Wallis test with Dunn’s *post hoc* correction. *p < 0.05, **p < 0.01, ****p < 0.0001.

It was shown previously that antibodies to AnAPN1 (UF6b) can reduce transmission of *Plasmodium* in a mosquito (36). Purified total IgG from treatment groups 25UF-0.25C and 5UF+AddaVax™ was tested by SMFA (**Figure 3A**) at a concentration of 750 µg/ml, corresponding to 0.52 µg/ml and 2.4 µg/ml of antigen-specific IgG, respectively. At this concentration, we found that antibodies purified from group 5UF-AddaVax™ significantly reduce mean oocyst intensity by 80–90% across three independent replicates (**Figures 3Bi-iii**). Antibodies at the same total IgG concentration from 25UF-0.25C conferred between 62–65% reduction in oocyst intensity in two of the three replicates. These results demonstrate that PLGA-*b*-PEG mediated presentation and delivery of UF6b and CpG cues at experimentally determined optimal dose and antigen:adjuvant ratio to the local draining lymph nodes induces potent transmission-reducing activity against *P. falciparum*, suggesting that this platform is a promising approach for potentiating further an already promising malaria TBV candidate.

## Discussion

The UF6b construct, a dimer of a subdomain of the mosquito midgut protein, AnAPN1, was shown to focus the immune system response away from non-protecting immunodominant epitopes and towards neutralizing epitopes (8). To boost this neutralizing antibody titer, a biodegradable PLGA-*b*-PEG NP vaccine was leveraged to deliver antigenic (UF6b) and adjuvant (CpG ODN ISS 1018) stimuli directly to the lymph node as previously demonstrated and shown in this work (**Supplemental Figure 2**) (20, 21). For ease of manufacture, UF6b-NPs and CpG-NPs were fabricated separately and then mixed to obtain the UF6b and CpG doses and ratios of interest. This NP formulation strategy is anticipated to increase scalability while reducing costs in the future. Given that the size and surface charge of the NPs are similar, the drainage kinetics, lymph node distribution, and APC targeting and uptake will be similar *in vivo* (18). To skew the response to a Th2 antibody-mediated response, we used lower densities of antigen and CpG adjuvant (**Supplemental Table 2**: 1.686 UF6b mg/m^2^ and 0.423 CpG mg/m^2^), which was shown to give an enhanced Th2-polarized response in similar polymeric vaccine delivery systems (34, 35). In contrast, high densities of surface-conjugated antigen on NPs in HIV models were shown to lead to direct B-cell receptor engagement and robust antibody elicitation, suggesting that high antigen densities for targeting B-cells directly, instead of APC targeting, is of interest in future applications for further boosting of antibody response (37, 38). With respect to nanovaccine production (**Figure 1**), the conjugation efficiency and low batch-to-batch variability of the NPs demonstrates the feasibility of this platform for scalable production of nanovaccines (**Supplemental Table 1**) for malaria and other infectious diseases. The NPs were of uniform size in the optimal size range for lymph-node targeting (**Figure 1**) and, with optimized lyophilization conditions, were stable in -20°C storage for 3 months (**Supplemental Figure 1B**). While there was a statistically significant increase in size between the pre-lyophilized NP and the 1- and 3-month lyophilization time points, the nanoparticle size is still below the threshold size necessary for lymphatic drainage as previously reported (20) and therefore remains an acceptable lymph node-targeting modality for TBV candidate antigens such as AnAPN1 (UF6b).

The UF6b-NPs yielded a peptide 9-dominant response when delivered in conjunction with either adjuvant, while there was very little response to the peptide 7 neutralizing epitope in all groups tested (**Figures 2A–C**). This is similar to that observed for UF6b peptide mixed with AddaVax™, GLA-LSQ, or Alhydrogel™ adjuvants (8, 36). Although, testing a pre-immune pooled IgG control was not possible due to animal study protocol limitations, the likelihood that the transmission-reducing activity here is due to chance or the background presence of IgG is unlikely given the extensive studies conducted by multiple laboratories, including our own (6–8, 31–33, 36). Importantly, it was previously shown that high concentrations of monoclonal antibodies to non-functional epitopes on AnAPN1 (6), or pooled naïve sera or purified IgG from non-human primates and mice (36) do not have any intrinsic, non-specific transmission-reducing activity, as also demonstrated through direct feeding experiments on *P. berghei* infected control mice following immunization or by passively transferring anti-AnAPN1 antibodies (7, 39). Clearly, target antigen selection and antibody specificity are important, as not every midgut antigen is necessarily involved in malaria parasite infection of the mosquito and inordinately high concentrations of antibodies to these targets do not confer transmission-reducing activity (40). It has also been estimated that *P. falciparum* infection even in control groups could result in 10-18% of the mosquitoes remaining uninfected (33), but this would be equivalently represented in treatment groups as well receiving antibodies with or without any transmission-reducing activity. We observed 80-90% reduction in median oocyst numbers following treatment, consistent with all previous studies conducted with this TBV antigen across different *Plasmodium* transmission study models (6–8, 36). It is unclear if changing the presentation of peptide 7 (*e.g.*, cysteine residue to C terminus or flipping the order of peptide 9/peptide 7 on UF6b construct) will alter our attempts to focus the immune response to the peptide 7 neutralizing epitope or combination thereof. It may very well be that peptide 7 is not a potent epitope in the murine model as suggested by earlier studies (36). NPs and ordered orientation of proteins were used previously to display and immunofocus towards specific neutralizing epitopes in both HIV and influenza (41, 42). This ordered presentation of the UF6b construct and constituent peptide 7 and 9 epitopes on NPs would likely yield even greater neutralizing ability and potency of our nanovaccine and therefore warrants additional investigation. The best formulation 25UF-0.25C yielded similar humoral profile ratios of IgG1, IgG2a, and IgG2b subclasses as 5UF-AddaVax™ at Day 70 (**Figure 2D** and **Supplemental Figure 4**). Given that AddaVax™ adjuvant has been shown to elicit both a Th1 and Th2 response and the humoral profile of the 25UF-0.25C NP is similar, this suggests a similar mode of action and Th1/Th2 polarization elicited by the 25UF-0.25C NP group.

We observed that the addition of CpG-NP increased the speed and magnitude of the antibody response in a dose-dependent manner (**Figures 2F–G**) similar to our previous observation using the model antigen ovalbumin (21). For the 5 µg UF6b dose, there was a dose-dependent increase in antibody response magnitude between the 5UF-0.05C and 5UF-0.5C groups, with little difference between 5UF-0.5C and 5UF-1C groups, suggesting that there is a saturation point for boosting with CpG-NP (**Figure 2F**). The AddaVax™ adjuvant yielded a faster antibody development and higher total magnitude compared to 5UF-1C, suggesting that the CpG-NP as currently formulated cannot yield as strong of a response even with higher CpG : UF6b ratios given saturation. On the other hand, the 25UF-0.25C group had a slightly higher magnitude and speed of response compared to the 25UF-0C group, suggesting that either more CpG-NP was necessary to boost the response or that the 25 µg UF6b dose cannot be greatly improved upon using CpG-NP (**Figure 2G**) as UF6b is already a highly immunogenic antigen. Interestingly, the 5UF-1C group had similar magnitude in response to the 25UF-0C group after Day 40, suggesting that the CpG-NP at sufficient levels can greatly spare UF6b dose (5× dose reduction). Dose-sparing has profound importance in developing a malaria vaccine for roll-out in developing nations, where costs per vaccine dose remains an important factor.

Given that adjuvant and antigen cues need to be in the same endosome/lysosome compartment to generate an appropriately skewed antigen-specific response, CpG-1018 conjugation onto the same NP with UF6b may allow for further dose sparing or more potent adjuvant effect. Lower doses of mixed UF6b-NP and CpG-NP would result in NPs with the antigen and adjuvant cues being in separate endosome/lysosome compartments, resulting in lower vaccine efficacy. The conjugation of antigen and adjuvant cues on the same NP surface with a 100:1 UF6b:CpG or higher ratio requires additional investigation (43). The influence of inoculation site on the resulting immune response is well known. Although we noted that the humoral response’s IgG subclass signature is similar to AddaVax™ adjuvanted group, a lower magnitude of overall response was observed. However, the contribution of a potent antigen depot effect of UF6b-NP : AddaVax™ at the inoculation site (since AddaVax™ is a squalene-based oil-in-water nano-emulsion formulated to mimic the Novartis MF59^®^ adjuvant) cannot be ruled out. These data point to considerations in the future for co-inoculation of UF6b-NP/CpG-NP for direct priming of the lymph node and UF6b-NP : AddaVax™ (or non-NP modified UF6b) for depot antigen presentation *in situ*.

In this work, we found that all Nano-UF6b particles, either formulated with or without Nano-CpG, induced a potent humoral immune response in CD1 outbred mice. Here, we chose CD1 outbred mice to account for genetic variation that is more representative of the diversity in a human population compared to the relatively homogeneous BALB/c inbred mouse model. Furthermore, we demonstrated that delivery of UF6b-NP and CpG-NP results in elicitation of a predominantly peptide 9-specific antibody response. The CpG-NP influenced the rapidity and magnitude of the humoral immune response, albeit to a limited extent, potentially due to the innate strong immunogenicity of the UF6b construct. Finally, the antibodies elicited reduced parasite development in the mosquito with the 25UF-0.25C NP group having similar neutralizing potency as AddaVax™. Given that NP delivery of antigen and adjuvant allows for more efficient colocalization of cues to individual dendritic cells, we utilized lower doses of CpG-1018 ISS than what is seen in the clinic. Our study enabled a quick bracketing of UF6b and CpG doses. It is anticipated that a 100:1 ratio at 50 µg UF6b and 0.5 µg of CpG will be the ideal fixed dose/ratio, which will ensure an earlier, higher, and broader humoral immune response at 4 weeks following only a single priming dose. Together these results show that the UF6b construct delivered to the lymph node using the PLGA-*b*-PEG nanovaccine delivery system elicits functional, transmission-reducing antibodies using less antigen and underscores that higher antibody titer may not necessarily be the correct target in vaccine design. The higher elicited functional antibody titer from nanoparticle delivery suggests that presentation of antigen is critical. This novel PLGA-b-PEG mediated delivery and presentation of antigenic and adjuvant cues to the lymph node warrants additional investigation in larger animal models.

## Data Availability Statement

The raw data supporting the conclusions of this article will be made available by the authors, without undue reservation.

## Ethics Statement

The animal study was reviewed and approved by Institutional Animal Care and Use Committee of Johns Hopkins University, Institutional Animal Care and Use Committee of University of Florida, and Institutional Animal Care and Use Committee of University of North Texas Health Science Center.

## Author Contributions

Conceptualization, GH, H-QM, and RD. Methodology, GH, NB, PK, VN, TH, WW, JS, and BL-G. Formal analysis, GH, PK, VN, and NB. Data curation, GH, NB, and PK. Writing – original draft preparation, GH and PK. Writing – review and editing, GH, NB, PK, BL-G, VN, TH, H-QM, and RD. Supervision, H-QM and RD. Funding acquisition, H-QM and RD. All authors contributed to the article and approved the submitted version.

## Funding

This work was funded by support from the National Institutes of Health (R01 AI144609 and U01 AI155361), National Science Foundation Graduate Research Fellowship Program (DGE174891), and funding from the University of Florida (UF) Preeminence Initiative through the UF College of Veterinary Medicine and the UF Emerging Pathogens Institute. Support was also provided by University of North Texas Health Science Center (UNTHSC) Preclinical Services group.

## Conflict of Interest

The authors declare that the research was conducted in the absence of any commercial or financial relationships that could be construed as a potential conflict of interest.

## Publisher’s Note

All claims expressed in this article are solely those of the authors and do not necessarily represent those of their affiliated organizations, or those of the publisher, the editors and the reviewers. Any product that may be evaluated in this article, or claim that may be made by its manufacturer, is not guaranteed or endorsed by the publisher.
