# Supplementary material for: Immunopotentiation by Lymph-Node Targeting of a Malaria Transmission-Blocking Nanovaccine
Source: Front Immunol. 2021 Aug 27;12:729086. doi: 10.3389/fimmu.2021.729086 (PMC8432939; doi:10.3389/fimmu.2021.729086)
Supplement: Supplementary file 1 [file DataSheet_1.pdf]

frontiers

Supplementary Material

Supplementary Tables

Supplemental Table 1.
UF6b-NP and CpG-NP Batch-to-Batch Consistency

NP Group	Batch	Number Average Diameter	Z-Average Diameter	Uniformity	Zeta Potential	Conjugation Efficiency
Cargo-NP	*#*	*nm*	*nm*	*PDI*	*mV*	*%*
Mal-NP	N/A	29.9 ± 1.3	53.4 ± 1.4	0.22 ± 0.03	-8.6 ± 0.2	N/A
UF6b-NP	1	30.0 ± 3.2	54.3 ± 0.44	0.20 ± 0.03	-7.4 ± 0.4	86.8%
UF6b-NP	2	32.1 ± 2.0	54.8 ± 0.9	0.21 ± 0.01	-7.6 ± 1.1	81.0%
UF6b-NP	3	30.4 ± 0.8	54.8 ± 0.8	0.20 ± 0.01	-6.9 ± 0.5	91.3%
UF6b-NP	4	30.4 ± 1.6	54.3 ± 0.2	0.18 ± 0.02	-6.8 ± 0.6	89.1%
CpG-NP	1	32.7 ± 4.2	54.1 ± 4.5	0.24 ± 0.07	-15.1 ± 1.7	51.7%
CpG-NP	2	31.5 ± 3.8	52.6 ± 1.9	0.21 ± 0.04	-13.5 ± 0.6	57.1%
CpG-NP	3	31.3 ± 1.3	55.2 ± 3.5	0.26 ± 0.05	-14.7 ± 0.5	51.3%
CpG-NP	4	30.7 ± 2.2	54.0 ± 2.2	0.25 ± 0.05	-16.1 ± 0.6	53.0%

*Zeta potential measured in 10 mM HEPES 10 mM NaCl (pH 7.4)

Note: Four separate nanoparticle batches were made and characterized to demonstrate the reproducibility of the UF6b and CpG conjugation method to the surface of functionalized PLGA-*b*-PEG-Maleimide (Mal-NP) nanoparticles.

Supplemental Table 2.
UF6b-NP and CpG-NP Antigen/Adjuvant Surface Density and Valency of CpG and UF6b on PLGA-*b*-PEG nanoparticles

Group	Surface Area	Density	Valency
Cargo-NP	*m^2*	*mg/m^2*	*molecules/NP*
UF6b-NP	$2.83{*}10^{-15}$	1.686	100.7
CpG-NP	$2.83{*}10^{-15}$	0.423	100.7

Supplementary Figures

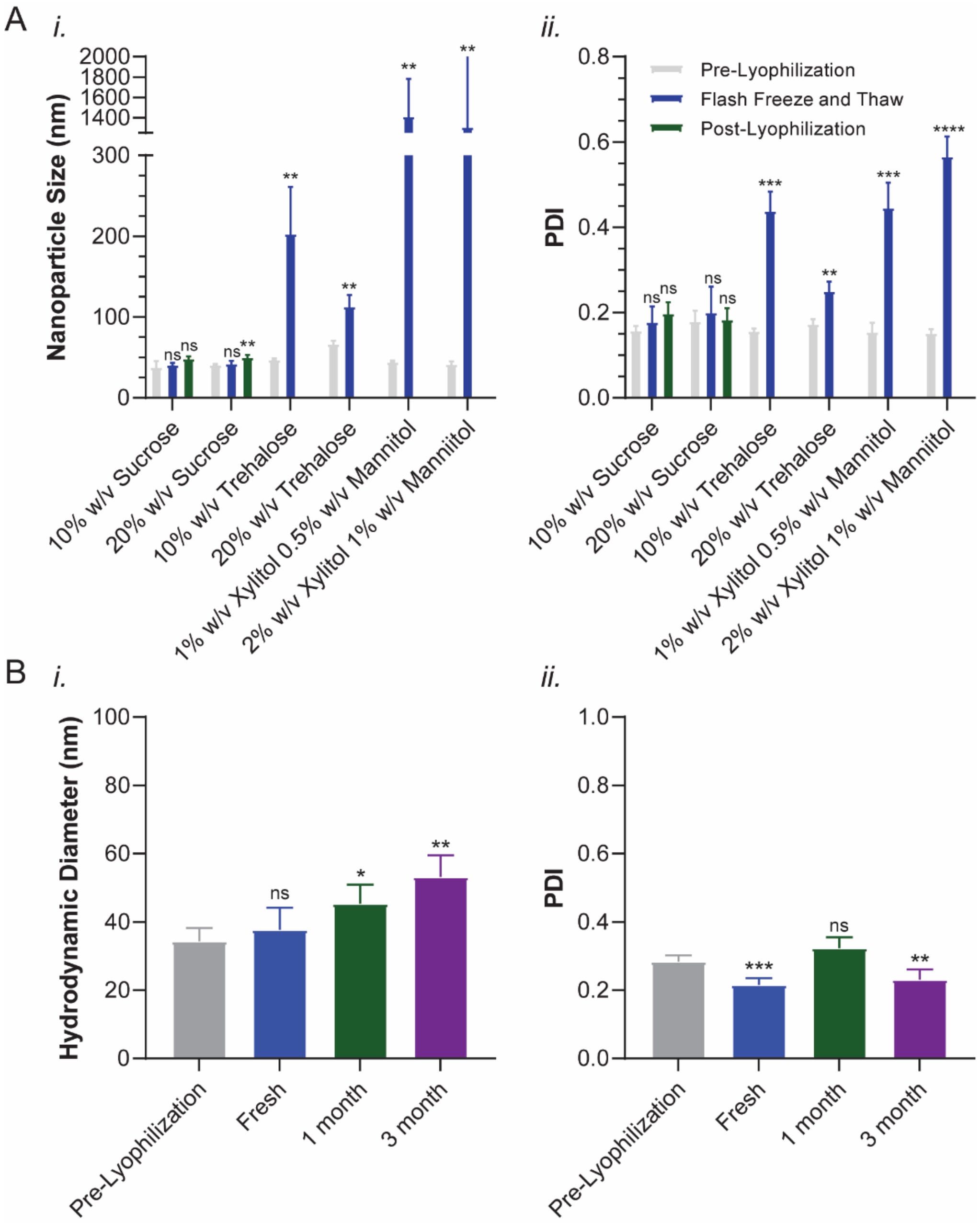

Supplemental Figure 1. Cryoprotectant selection and lyophilization stability of NPs.
(A) Lyophilization of unconjugated 1.5 mg/mL PLGA-b-PEG nanoparticles in sucrose, trehalose, or xylitol/mannitol were screened using a flash-freeze and thaw and then a flash-freeze and lyophilization protocol. The (*i.*) number average size and (*ii.*) polydispersity were measured.
(B) Using the 25UF-0.25C group as a test case, nanoparticles were lyophilized in 10% w/v sucrose and then reconstituted immediately post-lyophilization (fresh), 1 month, and 3 months. The (*i.*) number average size and (*ii.*) polydispersity were then measured by dynamic light scattering. Error bars represent standard deviation of three samples. Statistical significance was analyzed using ANOVA followed by Tukey's multiple comparison test or unpaired t test for direct comparison for comparisons between two groups. All groups were compared to the Pre-Lyophilization control.

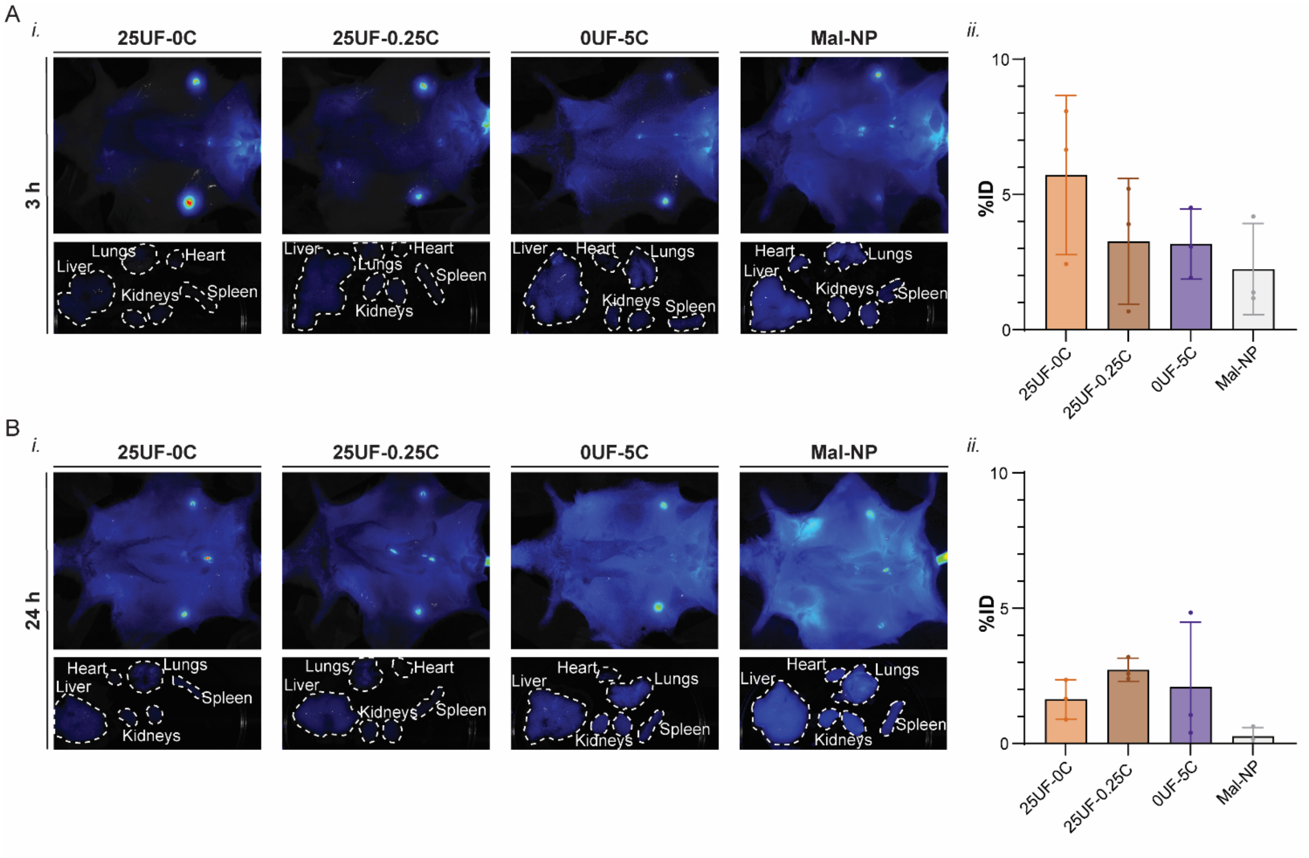

Supplemental Figure 2. Nanoparticle whole-body imaging of treatment biodistribution after *s.c.* administration. Biodistribution of each nanoparticle treatment including 25 µg UF6b-NP + 0 µg CpG-NP (25UF-0C), 25 µg UF6b-NP + 0.25 µg CpG-NP (25UF-0.25C), 0 µg UF6b-NP + 5 µg CpG-NP (0UF-5C), and unconjugated maleimide nanoparticle (Mal-NP) (*i.*) are shown. The signal from (*i.*) were quantified to determine the percentage of injected dose (%ID) in the inguinal lymph node compartment after (A) 3 h and (B) 24 h post *s.c.* administration is shown (*ii.*). All nanoparticle treatments demonstrate lymph node drainage, specifically to inguinal lymph nodes and some drainage to major reticuloendothelial system (RES) organs. Representative images of Pearl Imager whole body imaging. Data represent mean of triplicates and error bars indicate standard deviation.

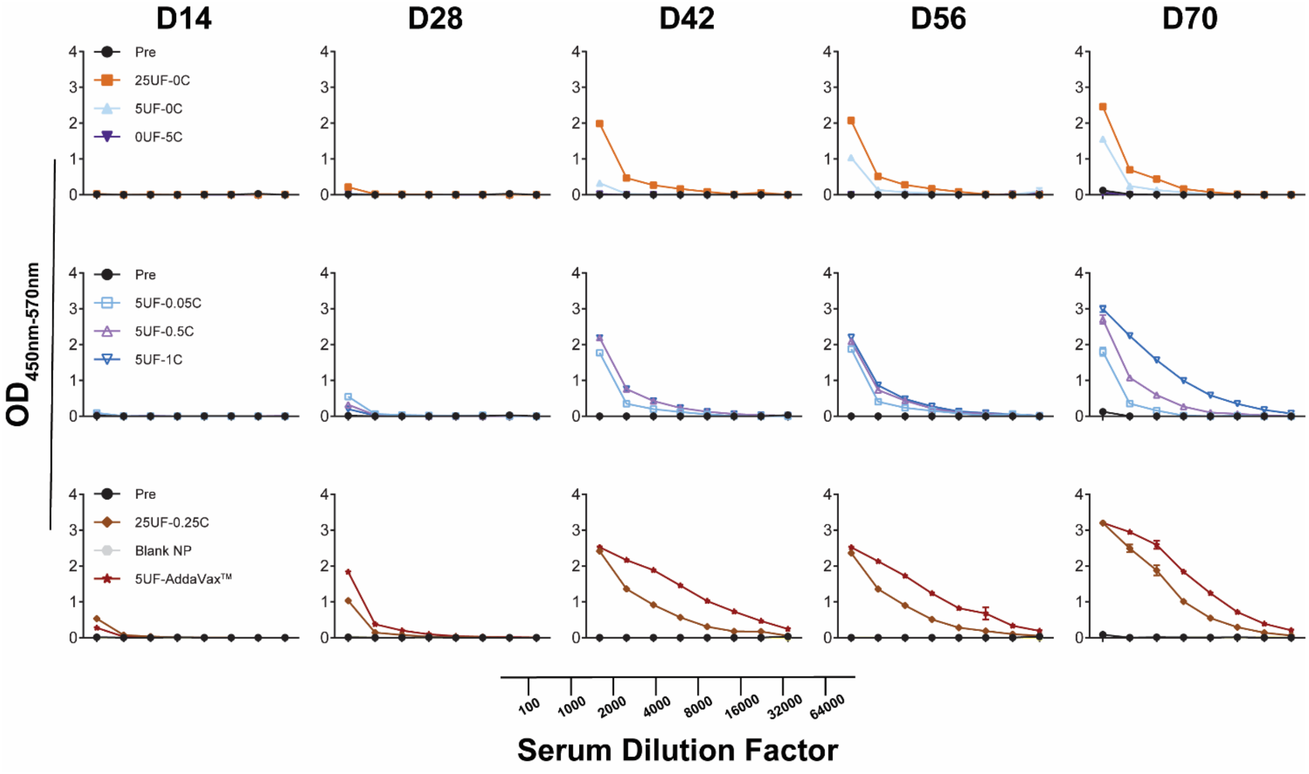

Supplemental Figure 3. Nanovaccine anti-UF6b antibody kinetics over 70 days. CD1 mice were immunized twice, two weeks apart, *s.c.* with the following formulations: Blank NP, 5 µg UF6b-NP + 0 µg CpG-NP (5UF-0C), 5 µg UF6b-NP + 0.05 µg CpG-NP (5UF-0.05C), 5 µg UF6b-NP + 0.5 µg CpG-NP (5UF-0.5C), 5 µg UF6b-NP + 1 µg CpG-NP (5UF-1C), 25 µg UF6b-NP + 0 µg CpG-NP (25UF-0C), 25 µg UF6b-NP + 0.25 µg CpG-NP (25UF-0.25C), 5 µg UF6b-NP + AddaVax™ (5UF-AddaVax™), or 0 µg UF6b-NP + 5 µg CpG-NP (0UF-5C). Graph shows mean values for each group (rows) at each time point (columns). Pooled sera from 6/mice per group were analyzed. Data points represent mean of triplicates. Error bars indicate SEM of triplicates, although in some cases the variation is tightly controlled, and the error bar is obscured by the data point.

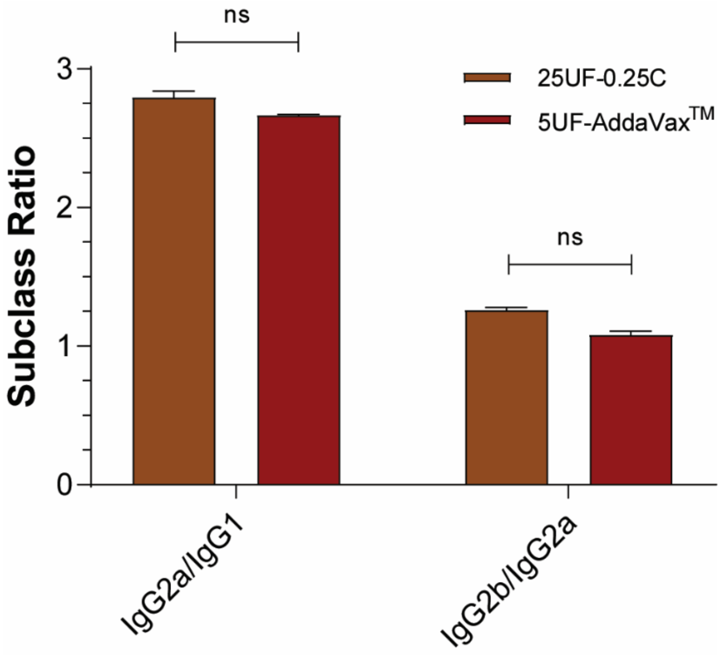

Supplemental Figure 4. IgG antibody subclass ratios. Humoral profile of the 25UF-0.25C and 5UF-AddaVax™ assessed by ratios of IgG subclasses using D70 sera at 1:100 dilution. Mean subclass ratios represent mean of duplicates with error bars indicating standard deviation. Statistical significance was determined by unpaired *t*-test for direct comparisons.

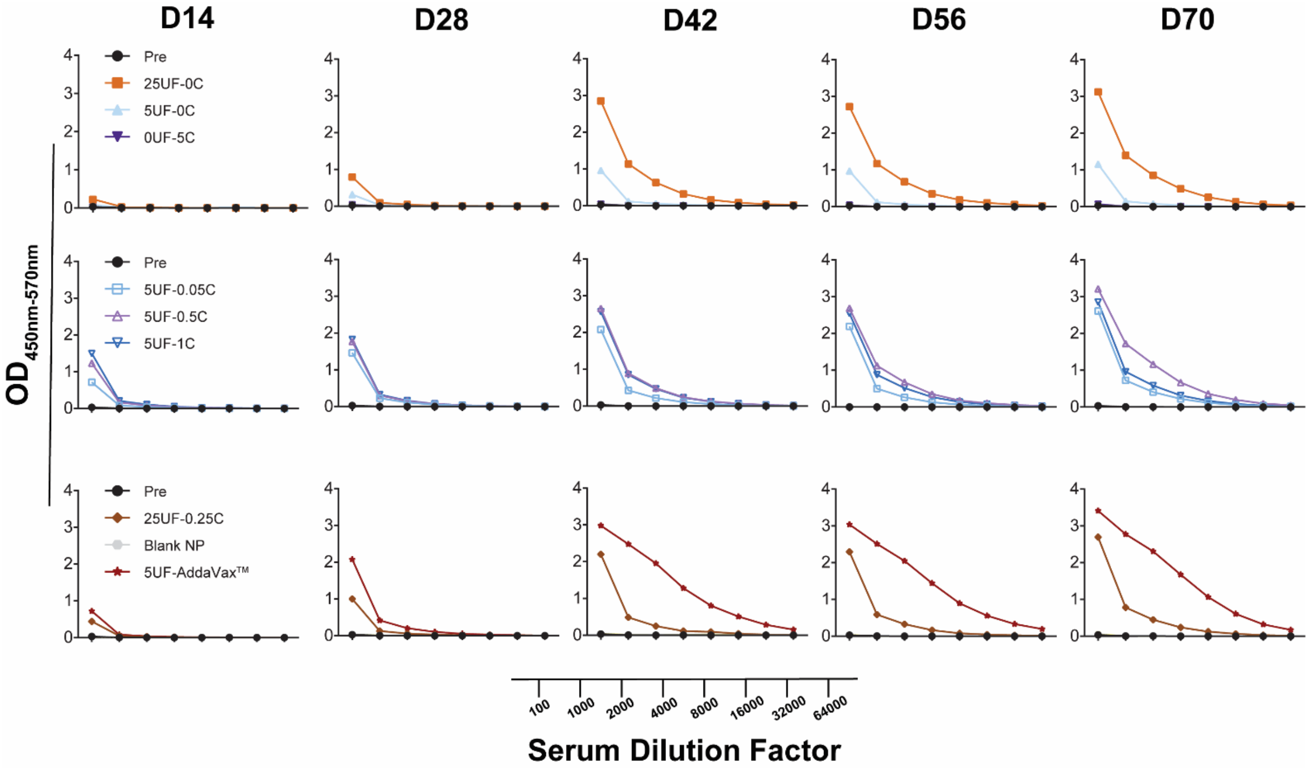

Supplemental Figure 5. Nanovaccine anti-UF6b antibody kinetics over 70 Days, second cohort replicate. CD1 mice were immunized twice, two weeks apart, *s.c.* with the following formulations: Blank NP, 5 µg UF6b-NP + 0 µg CpG-NP (5UF-0C), 5 µg UF6b-NP + 0.05 µg CpG-NP (5UF-0.05C), 5 µg UF6b-NP + 0.5 µg CpG-NP (5UF-0.5C), 5 µg UF6b-NP + 1 µg CpG-NP (5UF-1C), 25 µg UF6b-NP + 0 µg CpG-NP (25UF-0C), 25 µg UF6b-NP + 0.25 µg CpG-NP (25UF-0.25C), 5 µg UF6b-NP + AddaVax™ (5UF-AddaVax™), or 0 µg UF6b-NP + 5 µg CpG-NP (0UF-5C). Graph shows mean values for each group (rows) at each time point (columns). Pooled sera from 6/mice per group were analyzed. Data points represent mean of triplicates. Error bars indicate SEM of triplicates, although in some cases the variation is tightly controlled, and the error bar is obscured by the data point."

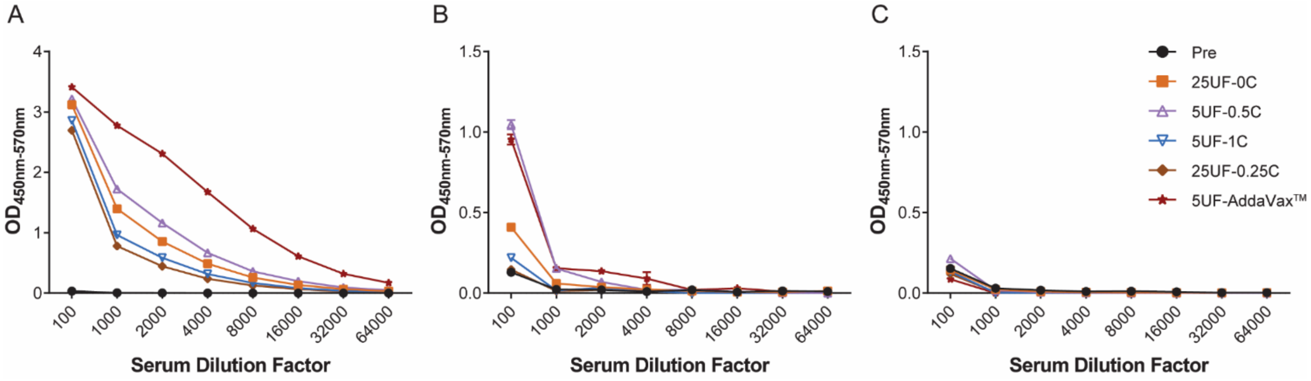

Supplemental Figure 6. Specificity of antibody to whole UF6b or constituent peptide 7 or 9 sequences, second cohort replicate. (A) Antibody-specific titer to whole UF6b peptide construct. (B) Antibody-specific titer to peptide 9 sequence. (C) Antibody-titer specific to peptide 7 sequence. All data is from day 70. Pooled sera from 6/mice per group were analyzed Data points represent mean of triplicates. Error bars indicate SEM of triplicates, although in some cases the variation is tightly controlled, and the error bar is obscured by the data point
